# Vaccines for Perinatal and Congenital Infections—How Close Are We?

**DOI:** 10.3389/fped.2020.00569

**Published:** 2020-12-15

**Authors:** Tulika Singh, Claire E. Otero, Katherine Li, Sarah M. Valencia, Ashley N. Nelson, Sallie R. Permar

**Affiliations:** ^1^Duke University Medical Center, Duke Human Vaccine Institute, Durham, NC, United States; ^2^Department of Molecular Genetics and Microbiology, Duke University, Durham, NC, United States

**Keywords:** vaccines, immunology and infectious diseases, congenital infections, perinatal infections, child health

## Abstract

Congenital and perinatal infections are transmitted from mother to infant during pregnancy across the placenta or during delivery. These infections not only cause pregnancy complications and still birth, but also result in an array of pediatric morbidities caused by physical deformities, neurodevelopmental delays, and impaired vision, mobility and hearing. Due to the burden of these conditions, congenital and perinatal infections may result in lifelong disability and profoundly impact an individual's ability to live to their fullest capacity. While there are vaccines to prevent congenital and perinatal rubella, varicella, and hepatitis B infections, many more are currently in development at various stages of progress. The spectrum of our efforts to understand and address these infections includes observational studies of natural history of disease, epidemiological evaluation of risk factors, immunogen design, preclinical research of protective immunity in animal models, and evaluation of promising candidates in vaccine trials. In this review we summarize this progress in vaccine development research for Cytomegalovirus, Group B Streptococcus, Herpes simplex virus, Human Immunodeficiency Virus, Toxoplasma, Syphilis, and Zika virus congenital and perinatal infections. We then synthesize this evidence to examine how close we are to developing a vaccine for these infections, and highlight areas where research is still needed.

## Introduction

Congenital and perinatal infections are caused by pathogens that infect a pregnant woman and can be passed to the fetus during pregnancy by infecting and crossing the placental barrier or infecting the newborn during delivery in the birth canal. By definition, these infections are different from neonatal and childhood diseases that are contracted after birth. When passed to the newborn, these infections can lead to developmental defects, physical deformities, and lifelong disability. Maternal infection of the placenta may also lead to complications in pregnancy such as intrauterine growth restriction, miscarriage, and stillbirth. Given immunological and biological alterations that occur in pregnancy, the course of infection and immunity can be more severe in pregnancy, which dually impacts maternal health and ability to recover. Moreover, pregnancy is a time during which many treatments may be contraindicated, which limits options upon diagnosis. In these settings, vaccines are a valuable tool to prevent congenital infections. While there are three licensed vaccines to prevent congenital infections, many more are in development.

In this review, we outline key strategies for vaccine design to prevent congenital and perinatal infection and highlight the three licensed vaccines. Then we detail vaccine development efforts, protective immunity, and design considerations for congenitally transmitted pathogens that threaten the health of newborns: Cytomegalovirus, Group B Streptococcus, Herpes simplex virus, Human immunodeficiency virus, Toxoplasma, Syphilis, and Zika virus.

## Strategies for Vaccines to Prevent Congenital Infections

There are two broad strategies for immunization to prevent congenital infections: (1) vaccinating women prior to pregnancy to generate protective maternal immunity; (2) providing passive or active immunization during pregnancy to boost immunity and prevent congenital transmissions with possible exposures later in pregnancy or at delivery. Vaccines against congenital infections depend on eliciting protective immune responses against the congenitally transmitted pathogen, that are sustained at a protective level at least through pregnancy. A protective threshold of antibodies or immune responses is ideally required prior to pregnancy to prevent maternal infection, and thereby prevent congenital transmission. Therefore, defining immune correlates is critical to the design and development of vaccines to prevent congenital infections.

For vaccinations delivered before pregnancy, high rates of seroconversion across populations and long-lasting immunity are critical features. As with the Rubella vaccine provided in childhood, vaccine-elicited immunity must protect diverse populations of women during a wide range of ages when they may be pregnant. As most pregnancies are unplanned, the optimal vaccine to prevent congenital infections must be administered well before the earliest age groups for women's reproductive age.

Importantly, active and passive vaccine-based strategies work together with other measures that support in preventing and controlling congenital infection. Infant health can also be improved if a worse secondary disease can be prevented, after the congenital infection or exposure. For example, with perinatal Hepatitis B exposure, a birth dose of vaccine and immunoglobulin protects the newborn from liver disease over their lifetime. Administration of drugs after birth is a secondary prevention measure intended to reduce morbidity and mortality by preventing disease progression after exposure. Moreover, there are complementary measures to reduce risk of transmission and severity of disease such as maternal or neonatal treatments, and public health measures to reduce exposure during pregnancy by limiting population-wide transmission. Specifically, these include programs to promote condom use or control mosquitoes, depending on the route of transmission. Though there are guidelines to screen for disease early in pregnancy, implementation of TORCH pathogen screening and standard of care vary widely. In low resource settings in particular, limited antenatal care access results in missed opportunities for prevention of congenital infections (1, 2). Systemic structural barriers and failures of prevention and treatment strategies indicate a need to complement interventions with vaccines to prevent congenital diseases.

There are three licensed vaccines for prevention of congenital infections: Rubella, Hepatitis B, and Varicella, which all use the strategy of immunization in childhood to elicit long lasting immunity prior to pregnancy, as well as provide passive immunization of the infant during pregnancy. These vaccines serve as successful examples that inspire and guide the many more in development.

### Rubella

Rubella infection in pregnant women, especially during the first trimester, can lead to serious fetal consequences including spontaneous abortion, infant death, and congenital rubella syndrome (CRS). CRS describes a range of birth defects including hearing impairment, cataracts, congenital heart disease, and neurological impairment (3). To prevent CRS, the measles mumps and rubella combined MMR vaccine is recommended as a two-dose series after 12 months of age (4). As a live-attenuated virus vaccine, the MMR vaccine is contraindicated for administration during pregnancy (4). In the pre-vaccine era, the US epidemic of 1964–65 resulted in 6,250 spontaneous abortions, 2,100 infant deaths at birth or soon after, and CRS in 20,000 infants, leading to deafness, blindness, and mental impairment (5). More recently, lack of high levels of vaccine coverage and concern for waning of immunity has led to prenatal screening of women and recommendations of immunization at least 1 month prior to conception in unvaccinated women who desire to become pregnant (6). The WHO regions of the Americas and Europe have achieved CRS elimination, whereas globally, vaccine coverage is suboptimal and 31% of countries have yet to introduce the vaccine and benefit from this protection (7).

### Hepatitis B

The vaccine for Hepatitis B virus (HBV) is offered as a three-dose vaccine starting at birth and has achieved 84% global coverage as of 2015 (8, 9). Chronically infected pregnant women may transfer the virus to their infant at delivery. Thus, HBV screening is recommended in pregnancy (8). For mothers with an active infection or immunity below the correlate of protection a combination of infant immunoglobulin prophylaxis, active vaccine and antiviral therapy is recommended (8). Since earlier infection in life leads to a greater likelihood of chronic liver disease, cancer, and death, intervention at birth after perinatal exposure is crucial. A birth dose of the vaccine reduces the risk for neonatal infection by 72% and up to 90% when combined with HBV immunoglobulin post-exposure prophylaxis (10, 11). If left untreated, 70–90% of perinatally infected infants develop chronic infection by 6 months (12). While passive immunoprophylaxis provides temporary protection, the vaccine offers longterm protection (13).

### Varicella

Varicella zoster-virus is the causative agent of chickenpox and shingles. The live-attenuated VZV vaccine is recommended for infants older than 12 months and is intended to reduce morbidity and mortality from childhood infection (14). The vaccine elicits lifelong protective immunity (14). Historically, the virus would infect nearly everyone by middle ages, with greatest incidence of disease in elementary school years with low rates of maternal infection (15). Rarely, maternal infection during pregnancy can lead to congenital varicella syndrome, which is characterized by limb hypoplasia, skin abnormalities, encephalitis, neurological impairment, and low birth weight (16). Prenatal assessment of women for varicella immunity and vaccination at least 1 month prior to conception is recommended, though this is not regularly performed or feasible in the event of unplanned pregnancies (14). If a pregnant woman lacks immunity, is exposed to the pathogen, or becomes infected, VariZIG, a human VZV immunoglobulin, is offered as post-exposure prophylaxis for the mother and pre-term neonate depending on exposure risk (17). VariZIG is a passive immunization strategy that is most effective within 96 hours of exposure but is also approved within 10 days of infection (17, 18). Thus, far, most countries have not adopted the childhood varicella vaccine, due to cost and concerns of incomplete coverage leading to greater risk of disease in pregnancy as compared to childhood (19).

## Special Considerations for Design of Maternal Vaccines

Many maternal vaccination strategies to date are aimed at providing fetal and infant immunity to these pathogens by transfer of protective antibodies (20–23). Transfer of IgG across the placenta into the fetal compartment during pregnancy may have a role in preventing congenital and perinatal infections (24). Antibodies of the IgG isotype begin to cross the placenta around gestational week 13 when the neonatal Fc receptor, FcRn, starts to be expressed on the placenta (25, 26). Many qualities of the IgG antibodies may contribute to the efficiency of transplacental transfer, including IgG subclass, antibody avidity, and gestational stage (27–29). Placental transfer of antibodies is most efficient for the IgG1 subclass, followed by IgG4, IgG3, and IgG2 with decreasing efficiencies (25). This is due to IgG glycosylation and varying binding affinities of the Fc region of the IgG subclasses to the FcRn (30, 31). Since IgG1 are elicited more in response to proteinaceous antigens than polysaccharide antigens, vaccine antigens can be designed to incorporate proteinaceous elements of the pathogen.

However, high titers of placentally transferred antigen-specific IgG in the infant may also reduce the magnitude of the infant's *de novo* immune responses to vaccines containing that antigen (32–34). This is best studied in the context of the measles vaccine, which is a live-attenuated replicating vaccine. While morbidity and mortality are reduced in children vaccinated against measles in the presence of maternal antibodies, protective neutralizing antibody responses are not established until booster doses when maternal antibody has waned (35, 36). This phenomenon, known as maternal antibody interference, and has been documented with many types of vaccines, including live-attenuated, inactivated, and protein or polysaccharide (subunit or conjugate) vaccines (37–49). Thus, vaccine design and timing must be guided by the requirements for IgG transfer to optimize prevention of congenital infection, as well as requirements for neonatal immunity prior to the age of greatest risk of exposure.

## Progress in Vaccine Development for Perinatal and Congenital Infections

For each pathogen, we detail transmission route, disease burden, evidence for current clinical guidance, key features of protective immunity, and vaccine design considerations. These crucial aspects are synthesized to evaluate progress and gaps remaining toward vaccine development.

## Cytomegalovirus (CMV)

*In utero* transmission of CMV is the most common congenital viral infection as ~1 in every 200 babies, or 30,000 infants annually, are born with congenital CMV (cCMV) in the US (50–54). While CMV infection is typically asymptomatic in healthy adults, including pregnant women, the major challenge for prevention of cCMV stems from viral latency, which allows CMV to persist and reactivate over a lifetime (55–57). In the most severe cases, vertical transmission of CMV can lead to fetal loss; more commonly, cCMV infection can cause severe defects and sequelae in the neonate, including hearing loss and developmental delays and which occur in an estimated 20% of cCMV-positive infants (53, 58, 59). This leads to lifelong disability. The risk of placental CMV transmission is greater for seronegative women who have primary infection during pregnancy (30–50%) than for chronically infected women experiencing secondary infection or viral reactivation (1–4%), indicating that the maternal adaptive immune response can be protective (50, 53, 60–63). However, reactivation of latent CMV or re-infection in seropositive pregnant women accounts for the majority of congenital infections because 60–90% of the global population is seropositive for CMV, with higher prevalence in developing countries (64–66).

CMV is shed in body fluids such as urine, saliva, breast milk, and semen and is typically transmitted via physical and mucosal contact with such fluids (50, 67–71). Consequently, direct contact, breast feeding, organ transplants, and blood transfusions are the possible routes of transfer in addition to congenital transmission from mother to the fetus. CMV can be transmitted vertically in any trimester of pregnancy, indicating that the target population for a vaccine to prevent cCMV would be women of child-bearing age prior to conception (72–78). The risk of transmission is greatest in the third trimester, but the risk of the child developing sequelae is greatest when transmission occurs early in gestation (61, 74, 79). People who spend a significant amount of time around young children, including childcare workers, teachers, and parents, are especially at risk. In high-income countries, maternal exposure through toddlers at daycare is a key route of exposure to the virus for pregnant women. As of now, there is no licensed vaccine for prevention of cCMV, despite over 40 years of research. The development of a CMV vaccine has been designated as a top priority by the National Academy of Medicine, and preclinical and clinical research efforts are on-going (80).

### Current Guidance

There is no clinical guidance to test for maternal CMV infection in the prenatal period. Because CMV infection in most adults is asymptomatic, cCMV infection during pregnancy is typically identified by abnormal fetal ultrasound findings, such as echogenic fetal bowel, cerebral ventriculomegaly, periventricular calcifications, and fetal growth restriction, which leads to maternal CMV testing (63, 77, 81, 82). Maternal infection is identified by detecting viral DNA via PCR in serum or urine or by assessing CMV-specific serum antibodies and avidity (63, 83). Congenital infection is determined via amniocentesis, generally only when maternal infection has been confirmed and after 21 weeks of gestation (63, 84–86). Testing for CMV at birth is becoming more commonplace, but traditionally, doctors will only order a CMV test if a baby shows multiple symptoms that can be associated with cCMV, such as low birth weight, jaundice, and/or microcephaly (50, 67, 87–90). However, CMV testing is recommended for newborns with documented sensorineural hearing loss regardless of the presentation of other symptoms or sequelae (89).

Several studies highlight the safety of administering hyperimmune globulin (HIG) treatment in pregnancy and benefits such as controlling maternal viral load, lowering rates of congenital transmission, and reducing severity of neonatal infection. Though the evidence remains divided as to the value of this intervention: two observational studies, a case-control, and a non-randomized study reveal that HIG lowered rates of vertical transmission, whereas two follow-up randomized placebo-controlled trials did not confirm this finding (91–95). More recent studies indicate that high-dose HIG and maternal DNAemia independently predict congenital transmission, suggesting that multiple factors should be considered for prevention of cCMV by HIG (96).

Though treatment options are currently limited, there is guidance for preventing CMV infection during pregnancy, including limiting new sexual partnerships and, more importantly, exposure to young children and their various bodily fluids. Given that exposure to urine and saliva from toddlers is an important route of transmission, pregnant women frequently exposed to young children are encouraged to take personal precautions such as washing their hands often, especially after changing diapers, and avoiding sharing food, drinks, and eating utensils with young children (67, 97–99).

### Protective Immunity

Animal models that have been employed to study placental CMV infection, include guinea pigs and non-human primates. Traditionally, the guinea pig model has been used to study cCMV infection because guinea pig CMV is capable of crossing the placenta in pregnant guinea pigs, while other small animals cannot model the placental viral transmission (100, 101). Pre-clinical vaccine candidates include vectored viral glycoproteins, glycoprotein B (gB) protein, the pentameric complex of CMV involved in cell entry, and combinations of surface proteins (102). These candidates show promising results in the guinea pig animal model with respect to reduction of maternal viral load and improved fetal health (103–107). Recently, a pregnant rhesus macaque model of cCMV following primary infection during pregnancy was developed. Early studies using this model found that CD4+ T cell depletion prior to challenge produces a consistent measurable phenotype of cCMV transmission (108, 109). In the absence of CD4+ T cells, passive infusion of potently neutralizing antibodies against rhesus macaque CMV (RhCMV) was protective against placental RhCMV transmission (110). This model promises to further our understanding of maternal protection against placental transmission of CMV as the rhesus macaque immune system better models that of humans and there is significant functional homology between the RhCMV and human CMV (109, 111, 112). Thus far, these studies indicate the importance of CD4+ T cells in a protective maternal CMV immune response and the protective effect of humoral immunity (108, 110, 113).

To date, the most effective CMV vaccine candidate tested in clinical trials is comprised of gB, one of the glycoproteins responsible for viral entry into cells upon infection (114), with a MF59 squalene adjuvant. This is currently the only preventative platform against viral acquisition that has completed phase II clinical trials (115). This vaccine was tested in cohorts of post-partum women, adolescent girls, and transplant recipients, and resulted in a partial efficacy of 50% against CMV acquisition as well as successfully prevented viremia in transplant recipients (115–117). Studies that stem from these trials continue to inform the immune correlates associated with protection from acquisition. While CMV neutralizing antibodies have been implicated in reducing placental transmission in the context of primary infection during pregnancy (103, 110), CMV neutralization titers did not correlate with protection in the gB/MF59 vaccine trials (118–124). Rather, non-neutralizing antibody effector functions, such as antibody-dependent cellular phagocytosis, have recently been implicated in mediating protection in the gB/MF59 vaccine trials, within the target population of adolescent girls and postpartum women (118, 124, 125). This suggests that non-neutralizing antibody effector functions may be an important part of CMV immunity in addition to neutralizing antibody responses.

Another vaccine strategy has been to target T cell responses to viral proteins in order to mediate robust viral clearance from tissues. This is supported in the context of congenital infection by the finding that CD4+ T cells are important for preventing placental transmission in the rhesus macaque model of primary infection during pregnancy (108). The focus of vaccine candidates tested in pre-clinical studies has been on pp65 and IE1, as they contain T cell epitopes present in many seropositive individuals (126–130). Pre-clinical studies using pp65 or homologs as the vaccine immunogen have yielded improved pregnancy outcomes, indicating that cellular immunity against tegument proteins can be protective in the context of congenital infection (128).

Neutralizing humoral immunity has been targeted throughout vaccine development efforts and is achieved by vaccinating against exposed glycoproteins (gB, gH, and pentameric complex) important for viral entry into the cell. Neutralizing antibody responses have been associated with reduced transmission during pregnancy in observational human cohorts as well as non-human primates (119–123). But neutralizing antibody responses were only minimally elicited in the partially protective gB/MF59 vaccine trial (118, 124). While single immunogen vaccinations are capable of producing both humoral and cellular immune responses, it is possible that a CMV vaccine must include multiple antigens to effectively activate protective neutralizing and non-neutralizing humoral immunity and cellular immunity (131). One pre-clinical study using an mRNA vaccine platform to deliver multiple antigens was able to elicit robust neutralizing antibody titers and T cell responses, showing promise for both the mRNA vaccine platform and a multi-antigenic approach to CMV vaccines (127, 132).

### Vaccine Design Considerations

The target population for a vaccine to prevent cCMV includes all women of child-bearing age, both seronegative women at risk of primary infection and seropositive women at risk of cCMV infection following re-infection or reactivation. While the optimal vaccine strategy would be effective in both groups, it may also be feasible to target the seropositive group alone as this group constitutes the majority of cCMV cases (133–135). Currently, the emphasis of vaccine developers is on women, and not men, because of the pregnancy-related transmission that vaccine efforts aim to prevent. Though men may facilitate transmission to pregnant women, the value of including men is unclear as of now.

A protective vaccine against cCMV must function in one or both of the following capacities: (1) prevent acquisition in seronegative mothers or reinfection in seropositive mothers; (2) reduce systemic viral load, infection of the placenta, and subsequent fetal infection (123, 136). Preclinical studies of protective immunity indicate that CD4+ T cells are critical to viral control in pregnancy and that neutralizing antibodies alone toward surface glycoproteins may be insufficient for prevention of cCMV. Thus, the ideal vaccine will likely need to activate a polyfunctional antibody response in addition to a timely CMV-specific cellular response to achieve protection (123, 130). Moreover, strain-specific differences in CMV may modulate vaccine immunity and further studies on vaccine design are required to evaluate the value of including viral components from multiple strains. For example, there are five genotypes of gB which form two phylogenetic supergroups, and vaccination with one genotype may induce adaptive responses that are protective against only a subset of CMV strains (137). These findings may also support the idea that multiple antigens or antigen genotypes are needed in an effective vaccine to prevent CMV infection.

### How Close Are We

A CMV vaccine is likely not as close as hoped due to limited knowledge of protective immunity, even after more than 40 years of research and high prioritization by the National Academy of Medicine. Greater opportunities for research and advocacy for universal CMV testing of neonates and identification of protective immunity are needed to be able to identify and treat congenital CMV and develop an efficacious vaccine.

## Group B Streptococcus

The gram-positive bacterium, group B *streptococcus* (GBS), colonizes the maternal vaginal tract and can lead to stillbirths and infection of the neonate by exposure during labor or an ascending infection of the amniotic fluid (138). Globally, 1 in 5 pregnant women are colonized by GBS, and 1% of all stillbirths are attributable to maternal GBS infection (139, 140). While only 1–2% of infants born to GBS colonized mothers have invasive bacterial disease, an estimated 10–50% of these cases are fatal (138, 141) Vertical transmission may lead to early onset of neonatal GBS disease in the first week of life, whereas late onset disease occurs in 7–90 days of life and is typically due to postnatal exposure to the pathogen from the colonized mother or the environment. Infant disease is characterized by sepsis, pneumonia, and respiratory distress, and in a subset, even meningitis. Of those infants who survive meningitis, 30–40% have neurodevelopmental impairments, leading to lifelong morbidity (142, 143). While peripartum intravenous antibiotic treatment with penicillin substantially reduces the incidence of early-onset disease, it does not confer any immunity to the newborn nor protect from late-onset disease (144). Also, some GBS isolates in the US and Japan have demonstrated decreased susceptibility to penicillin, indicating that antibiotic treatment may not be an adequate long term solution (145, 146). A vaccine is needed to protect infants from early as well as late onset GBS disease.

### Current Guidance

The CDC and WHO recommends that all pregnant women who are colonized with GBS should be treated with antibiotics during labor. The indication for antibiotic prophylaxis include a GBS-positive culture of a vaginal or rectal swab upon screening in late pregnancy, pre-term labor (<37 weeks), chorioamnionitis, prolonged labor, or ruptured membranes before labor (147, 148). This is because high levels of maternal colonization, fever, and prolonged rupture of membranes are risk factors for early-onset GBS disease in newborns (149, 150). With 98% of women receiving GBS testing in the US, incidence of early onset GBS has decreased to 0.25 per 1,000 (151, 152). Yet, there are several challenges to implementation of timely screening in low resource settings (153).

### Protective Immunity

Capsular polysaccharide (CPS)-specific maternal IgG levels correlate with protection from neonatal GBS disease. In the mouse model, infusion of human CPS IgG protected from challenge, indicating the antibodies are sufficient for mediating serotype-specific protection from disease (154, 155). Observational studies of natural immunity to GBS infection in the US, Europe, and South Africa indicate that the threshold for protection against early onset neonatal disease is maternal CPS-specific IgG in the range of 0.5–10 μg/mL (156–160). While the association between the magnitude of maternal IgG and neonatal protection is consistent across populations, further studies are needed to confirm an appropriate correlate of protection that can serve as an endpoint in vaccine trials. Specifically, standardization of CPS-specific IgG binding and functional assays, antigens, and reference sera will facilitate progress (161). GBS-binding antibodies mediate several functions; including opsonizing bacteria, direct complement mediated killing, and phagocytosis. These functions are cumulatively measured by *in vitro* opsonophagocytic assays. One study shows that maternal IgG concentrations >1 μg/mL facilitate GBS killing and reduce risk of early onset neonatal disease by 81 and 78% for serotypes Ia and III (158). In contrast to antibodies, CPS-specific immune cell activation is associated with clearance of homotypic GBS rectovaginal colonization during pregnancy, but not with the magnitude of CPS-specific IgG and opsonophagocytic activity, which are required for protection (162). Altogether these data indicate that antibodies are crucial to protection from GBS.

In order to protect newborns from early and late onset GBS, antibodies must be transferred across the placenta and remain at protective levels in the infant through 3 months of age, a time period of greatest risk of GBS disease. The concept of maternal immunization to promote transplacental transfer of IgG and protect neonates from GBS infection has been tested in baboons, mice, and rabbit models. These studies show increased survival in offspring of vaccinated animals upon challenge with serotype matched GBS, and functionally equivalent levels of antibody transfer from mother to offspring (163–165). Cumulatively, these studies support further development of glycoconjugate vaccines that elicit strong CPS-specific IgG in pregnancy for prevention of neonatal GBS.

### Vaccine Design Considerations

The leading vaccine candidates are CPS-protein conjugate vaccines. Specifically, the CPS conjugated to CRM197 (a non-toxic diphtheria toxin carrier protein) or tetanus toxoid, have been tested in Phase II vaccine trials as well as pregnant women. Since 5 GBS serotypes (Ia, Ib, II, III, IV) account for 97% of global neonatal GBS disease, vaccine candidates are designed to include one or multiple CPS antigens from these serotypes (166). Tetanus toxoid conjugate vaccines have been shown to be safe and immunogenic in non-pregnant as well as pregnant women (167–169) Antibody responses have been dose-dependent and elicited opsonophagocytic activity against matched GBS serotypes *in vitro* (167–169) In Phase II studies, administration of a second dose of a trivalent CPS-CRM197 vaccine candidate in non-pregnant subjects shows seroconversion with >8 μg/mL CPS type-specific IgG in >94% of participants for each serotype (Ia, Ib, and III). Antibody responses to glycoconjugate vaccine candidates peaked in their opsonophagocytic activity 4 weeks after immunization, declined substantially by 1 year, but persisted through 2 years (170). More recently, antibodies against the GBS surface proteins alpha and rib have been associated with less neonatal disease, suggesting that vaccines based on these conserved bacterial proteins may provide cross-serotype protection (171). These trials suggest that the glycoconjugate platform is effective immunogenic in the critical window of pregnancy.

Many features of disease and immunity support a maternal vaccine in pregnancy as the optimal strategy for prevention of neonatal GBS: (1) the risk to newborns is greatest in the days after birth, before a time when they may mount their own immune response; (2) transplacental transfer of antibodies at a protective level is feasible; (3) vaccine elicited immunity of current candidates is greatest in the weeks after immunization and wanes thereafter, supporting immunization immediately preceding the window of greatest risk to the newborn.

### How Close Are We

Due to the low neonatal disease incidence amongst live births (1–3 cases per 1,000), the sample size to test efficacy with the endpoint of reduced perinatal transmission may not be feasible (161). Therefore, identifying correlates of protection for each serotype that are consistent across countries will be a crucial pathway to vaccine licensure (172). What remains now is to clearly define the correlate of protection with further studies across populations and standardization of binding and functional antibody responses. With nearly 30 years of clinical trial data and strong safety and immunogenicity profile of vaccine candidates in pregnancy, a GBS maternal vaccine is feasible in the near future.

## Herpes Simplex Virus (HSV)

The prevalence of HSV types 1 and 2 among adolescents and young adults is 47 and 11% in the US, and type 2 is associated with genital herpes (173). Neonatal herpes infection affects ~every 1 in 3,200 births (174, 175). The majority (85%) of neonatal HSV infections are acquired peripartum during vaginal birth, 10% are acquired after birth from maternal mucosal virus shedding and 5% are acquired *in utero* (176). Evidence suggests that cesarean delivery is an option to reduce the risk of neonatal HSV, though not protective in all cases (177–179). Whereas, acyclovir administration in late pregnancy reduces viral shedding at delivery, impact on neonatal HSV has not been quantified (180, 181). Notably, risk of transmission is highest for pregnant women with a primary HSV infection during pregnancy (30–50%), as compared to seropositive women with recurrent HSV in pregnancy (1%) (177). These transmission rates indicate that pre-existing maternal immunity has a protective role in preventing vertical HSV transmission.

### Current Guidance

There are three patterns of HSV disease in newborns that can be identified within the first 3 weeks after birth. The first is localized cutaneous infection involving skin, eyes, and mouth observed in 45% of neonatal HSV infections (182). The second is infection of the central nervous system (CNS), which is observed in 30% of infants (182). The third is a disseminated infection that encompasses multiple organs in 25% of HSV-infected newborns (182). Though intravenous administration of 60 mg/day of acyclovir for 3 weeks has reduced infant mortality, ~29% of infants with disseminated disease and 4% of infants with CNS disease continue to die within the first year of life (183). Moreover, neonatal infection may lead to the development of a latent viral reservoir and reactivation of infection (184). Relapse of HSV infection can worsen disease and survival prognosis with conditions such as recurrence of skin lesions, infection of CNS, and severe neurologic and behavioral impairments (184–186). Though treatment has reduced infant mortality, preventative measures are needed to eliminate infant mortality due to congenital HSV and curb worsening neurologic pathologies resulting from reactivation of latent viral reservoir.

### Protective Immunity

Immune responses that protect against HSV infection are characterized by a robust tissue-resident T cell responses, which can lower viral burden in the genital tract and decrease transmission of infection to dorsal root ganglia, where HSV may establish latent infection and reactivate to cause recurrent disease later in life. It has been found that depleting T cells prior to HSV2 challenge in mice leads to more severe disease as compared to B cell depletion, indicating the prominent role of T cell responses (187, 188). Moreover, when HSV infection is not active, vaginal biopsies reveal increased levels of effector CD8+ T cells, indicating the importance of this cell type in viral control (189).

Maternal antibodies also have a role in supporting clearance of HSV infection and providing passive protection to the fetus. B cells and their corresponding antibodies reduce the time to resolving viremia upon infection as compared to B cell depleted mice, but passive transfer of antibodies alone does not prevent infection in naïve mice (187). Recent findings from murine models of maternal immunization indicate that protection of pups from passively transferred maternal antibodies is associated with antibody-dependent cellular cytotoxicity (ADCC), suggesting that ADCC-mediating antibodies may be more relevant to neonatal protection than neutralizing antibodies (190). Importantly, evaluation of intravaginal vaccines in the guinea pig and mouse models indicate that this vaccination route increases levels of IgG and IgA in the genital tract, the site of primary exposure to HSV (191, 192). These studies demonstrate that while HSV-specific antibodies can support viral clearance, they cannot alone prevent infection and recurrent disease.

There are several ways in which vaccines strive to elicit protective cell-mediated immunity. Recently a single replication HSV2 vaccine candidate with a deletion in the viral cell entry protein, glycoprotein D, was tested in mice and showed protection from lethal challenge (193). Intriguingly, this protection correlated with Fc receptor activating antibody titers and not neutralizing titers, reinforcing the importance of antibodies that facilitate immune cellular activity (193). While subcutaneous vaccination strategies have led to increased levels of circulating HSV-specific CD8+ T cells, this does not translate to higher levels of HSV-specific CD8+ T cells migrating to the genital tract, leading to ineffective protection and the need for novel strategies to elicit tissue-specific immunity (194). New vaccination approaches, such as the “prime and pull” strategy, are being developed to boost tissue-specific immunity. To test this strategy, mice and guinea pigs were immunized subcutaneously with attenuated HSV2 or a combination of HSV2 glycoproteins, and this “primed” systemic immune response was “pulled” toward the vaginal tract by topical application of toll-like receptor agonists (imiquimod) or chemokines that attract CD8+ T cells (CXCL9 and CXCL10) following vaccination (195, 196). This protected mice from lethal HSV2 challenge, and led to substantial increases in the magnitude of and longevity of HSV-specific CD8+ T cells in the vaginal tract as compared to subcutaneous vaccination alone (196). In addition to robust and lasting mucosal immunity, identifying appropriate topical products that “pull” the immune response, but do not cause unwanted inflammatory responses will be a key factor in the success of such innovative vaccine strategies (196). Altogether, these studies indicate that preventing maternal primary or secondary HSV infection requires robust mucosal immunity and long lasting HSV-specific tissue-resident CD8+ T cells.

### Vaccine Design Considerations

Despite progress in our understanding of protective immunity, vaccine candidates tested in guinea pig and mouse animal models have not translated to effective candidates in vaccine trials (197). There are several subunit and live-attenuated vaccine candidates that have been tested in vaccine trials (198). The Herpevac trials which contained the glycoprotein-D subunit of HSV2 demonstrated partial efficacy of 58% in protecting against HSV1 in seronegative women, and an overall efficacy of 20% against both HSV1 and HSV2 (199). Analysis of immune responses that led to protection in this subset indicated high neutralizing antibodies as a correlate of protection (200). Similarly, the Chiron HSV vaccine composed of HSV2 glycoprotein-B and glycoprotein-D subunits elicited neutralizing antibodies but failed to protect against HSV2 infection (201). These trials were halted due to low efficacy against HSV2 and indicate that neutralizing antibodies alone are insufficient in preventing primary infection, viral persistence, or recurrence of disease. Achieving greater efficacy will likely require a more robust cellular response and adjuvants tailored to the desired CD8+ T cell response.

Other platforms such as replication defective live-attenuated HSV2 (HSV529) and DNA vaccine encoding HSV2 glycoprotein-D (COR-1) are being tested in Phase 1 trials to improve cellular responses (202, 203). Thus far, these candidates are shown to be safe in humans, and the HSV529 elicits neutralizing antibodies and modest CD4+ T cell responses in HSV seronegative vaccinees (202). In addition, there are other subunit vaccines in preclinical development that are assessing combinations of different viral antigens with novel adjuvants to stimulate CD4+ and CD8+ T cell responses (204, 205).

### How Close Are We

The primary strategy to prevent congenital HSV is to immunize prior to conception to confer HSV immunity during pregnancy and reduce likelihood of reactivation and congenital transmission. In the absence of a vaccine that induces sterilizing immunity, even a partially effective vaccine that controls viral shedding could reduce congenital disease burden since isolation of HSV from the mother's vagina at delivery is associated with a 300-fold increase in the risk of neonatal infection (177). While the vaccines candidates tested thus far do not protect adequately from HSV2 acquisition, it is unclear if they would limit vertical transmission with differential efficacy, since analyses were conducted in non-pregnant populations. Testing of diverse vaccine candidates in clinical and pre-clinical trials gives hope that a vaccine to protect from perinatal HSV infection is feasible.

## Human Immunodeficiency Virus (HIV)

HIV can be vertically transmitted from mother to infant *in utero*, during delivery, or postnatally during breastfeeding. In 2018, ~1.7 million children worldwide under the age of 15 were living with HIV and over 160,000 infants were newly infected with HIV primarily via mother to child transmission (MTCT) (206). While HIV-infected infants could remain asymptomatic for years, HIV can progress to AIDS if left untreated, characterized by a severely damaged immune system with a CD4+ T cell count <200 and increasing frequency of severe, opportunistic infections. In the absence of interventions during or after pregnancy, rates of mother to child transmission (MTCT) are between 30 and 40% but the introduction of antiretrovirals therapies (ART) during pregnancy and breastfeeding has reduced MTCT rates to as low as 2% (207). Since the majority of HIV-positive individuals are started on ART following diagnosis, the primary observed impacts of HIV infection are typically related to the lifelong treatment. While ART inactivates replicating virus, the drugs cannot abolish the latent viral reservoir that results from infection, causing lifelong dependence on this drug. While ART in HIV-infected children prevents disease progression to AIDS, ART has been associated with metabolic complications including lipodystrophy, dyslipidemia, insulin resistance, lactic acidosis, and loss of bone density (208). Thus, additional options to support current prevention strategies against perinatal HIV transmission are needed to eliminate the pediatric HIV epidemic.

### Current Guidance

It is recommended that all pregnant women be tested for HIV during their first antenatal visit. Subsequent, testing in the third trimester is advised for pregnant women with negative initial HIV antibody tests if they are considered at increased risk of HIV acquisition, or reside in jurisdictions with elevated HIV incidence or that require third-trimester testing (209). According to the World Health Organization's (210) clinical treatment recommendations, all pregnant and breastfeeding women regardless of WHO clinical stage and CD4+ T cell count should receive the Option B plus treatment strategy, which consists of triple combination fixed-dose ART, immediately upon diagnosis and continued for life. A primary risk factor for MTCT of HIV is maternal plasma viral load, other risk factors include maternal disease progression, CD4+ T cell counts, mode of delivery, prematurity, and breastfeeding (211).

### Protective Immunity

In the absence of ART, the rate of MTCT of HIV is <50%, suggesting that there exist maternal immune factors that contribute to protection. Identification of these immune correlates of protection could inform the types of immune responses that maternal HIV immunization strategies may need to elicit to prevent transmission to an infant (24). Maternal antibodies against conserved portions of the HIV envelope (Env) have been suggested to have protective effects against transmission although the results have not been consistent across studies. Factors such as the magnitude of maternal IgG responses to the third variable loop or CD4 binding site of the HIV *env* gene, and the magnitude of neutralizing antibody responses against easy-to-neutralize (tier 1) viral variants were predictive of reduced risk of MTCT in the US-based Women and Infants Transmission Study (WITS) cohort (212). Whereas, increased risk of MTCT was associated with antibodies against CD4+ T cell binding sites in the HIV envelope and HIV's variable loop 1 and 2 (V1V2) (213). Moreover, the presence of gp41 epitope-specific antibodies is associated with reduced risk of transmission in HIV subtype C-infected mother-child pairs (214). These conflicting results indicate that while there are potentially protective humoral immune responses against HIV Env, these responses differ depending on virus clade, transmission mode, and maternal antiretroviral use (213, 215, 216).

Maternal autologous virus neutralizing antibody responses are another immune factor that has been increasingly studied in the context of MTCT of HIV. MTCT is characterized by a virus genetic bottleneck in which infant infection is established by a single or a few maternal transmitted-founder (T/F) viral variants (217–219). Though the maternal and infant factors that drive selection of infant T/F variants are not well-understood. Recent studies have found that infant T/F viruses are more neutralization resistant to paired maternal plasma neutralizing antibodies as compared to maternal non-transmitted viral variants (218). However, some studies have indicated that transmitting mothers as compared to non-transmitting mothers have higher breadth and potency of neutralizing antibody responses (220). These infant T/F viruses are generally neutralization sensitive to most broadly neutralizing antibodies (bnAbs). Yet, a recent study has revealed the occurrence of escape of the infant transmitted virus variant from a mother's plasma bnAb response, indicating that a single specificity bnAb response will not be sufficient to eliminate MTCT (221). Together, these studies suggest the potential for boosting maternal autologous virus neutralization by eliciting multispecific bnAb responses to further prevent MTCT (218).

Current research into passive and active immunization strategies for preventing MTCT is being conducted in both non-human primate (NHP) models and clinical trials. In the simian immunodeficiency virus (SIV) model, SIV-exposed infant macaques were protected against transmission by passive immunization with hyperimmune globulin (222). Rhesus macaque models utilizing chimeric simian-human immunodeficiency virus (SHIV), in which includes the HIV envelope, have also shown that post-exposure passive immunization of infants or mother-infant pairs with neutralizing monoclonal antibodies led to complete protection from virus acquisition (223, 224). Human clinical trials have tested treatment of HIV-infected pregnant women and infants with HIV hyperimmune immunoglobulin (HIVIG) in order to assess HIVIG safety and efficacy when combined with ART drugs like nevirapine and zidovudine (225, 226). While HIVIG administration had no adverse effects on pregnancy, there was no statistically significant difference in HIV perinatal transmission, showing that results from the promising NHP model did not translate to efficacy in humans (225, 226). Preliminary maternal HIV vaccination trials have established the safety and feasibility for delivery of viral envelope-based alum-adjuvanted vaccines in HIV-infected pregnant women on ART (227). Though the efficacy of this approach remains to be tested.

### Vaccine Design Considerations

A promising area of research is the development of a pediatric HIV vaccine that can elicit lifelong bnAb responses targeting locations on the HIV Env protein. Passive administration of bnAbs to NHPs can prevent transmission and establishment of viral infection, providing proof of principle that bnAbs of sufficient breath and potency could induce protective immunity (224). Current approaches to bnAb elicitation explore use of multi-component sequential regimens that incorporate HIV Env epitope-specific immunogens, antibody lineage-based immunogens, and/or germline-targeting immunogens to drive antibody maturation from B cell precursors toward bnAbs (228). Yet, these approaches are likely to require long term multi-dose, immunization strategies. BnAb targeting vaccines are of particular interest as a vaccination strategy to elicit long-term protective HIV immune responses in early childhood that will provide protection into adolescence, prior to the risk of sexual transmission. Recent studies have suggested that HIV-infected children are able to develop broader and more potent virus neutralization earlier than adults and via a distinct mechanistic pathway, highlighting potential advantages of the childhood immune landscape for eliciting broadly neutralizing antibodies compared to adults (229–232).

### How Close Are We

Despite more than 40 years of intensive research and investments in the field, HIV continues to be a challenging pathogen to address as it mutates away from vaccine immunity, presents highly complex epitopes, and consists of a spectrum of strains with high viral diversity. While clinical trials and the non-human primate model have revealed trends in immunity required to control the virus, these trends have varied across populations and viral strains leading to several hurdles for vaccine design (233). Promising strategies like the elicitation of bnAbs do not have a licensed precedent in medicine and have not been tested in clinical trials. Therefore, much research is still required to generate an effective vaccine for elimination of perinatal and lifelong infection with HIV.

## Toxoplasma

T*oxoplasma gondii* is a parasite prevalent in the world with up to one third of the world population infected (234). The percentage of women infected in child bearing years varies depending on the region. In developed countries, between 10 and 50% are infected, however in the tropics this rate can be as high as 80% (235). Such a high global prevalence of this parasite is facilitated by its partial life cycle in feline species, which co-inhabit with humans, ability to infect ubiquitous mammals and birds that we eat and live around, and ability to survive in the external environment without degradation. To do this, *Toxoplasma gondii* has a particularly complex life cycle with two main stages: the sexual life cycle which occurs in felines and results in oocysts that are transmitted in cat feces, and the asexual life cycle occurs in mammals or birds (236). The parasite takes different forms in each phase of its life cycle allowing it to adapt to each host and infect. The two life cycle phases that may progress in humans are known as, tachyzoites, which rapidly replicate, and the bradyzoites, which are the more inactive and form cysts in tissues (236). Not only do these cysts inhibit organ function, but they also contain concentrated amounts of parasites which facilitate vertical transmission. Consequently, the parasite is typically transmitted by ingesting contaminated undercooked meat and unwashed vegetables or fruit, and directly through handling and subsequent incidental ingestion of the parasite in cat feces or contaminated soil (234).

Infection results in the disease Toxoplasmosis. Most immunocompetent individuals have no symptoms (subclinical), however pregnant women and immunocompromised individuals such as HIV-infected individuals and transplant patients can have life threatening complications due to cysts and infection of organs like muscles, brain, heart, lung or placenta (237). Specifically for the fetus and newborn, toxoplasmosis can result in hydrocephaly, intellectual disability, seizures or death (237). Timing of congenital infection by trimester of pregnancy and severity of injury to the fetus are inversely related. During the first trimester of pregnancy infection results in a low transmission frequency (<6%) to the fetus but has the most detrimental consequences if transmission does occur (234). Whereas, infection in the third trimester transmission is much more common (60–80%), but results in lesser severity of disease in the fetus (234).

### Current Guidance

There is no vaccine for *T. gondii*, and in fact vaccines are not a high priority given effective treatment options in pregnancy and widespread prevention counseling in prenatal clinical visits. Prevention of Toxoplasma congenital infections is based on following proper hygiene guidelines during pregnancy, such as washing hands, avoiding eating raw or undercooked meat or unwashed vegetables, and importantly, not handling cat feces (238). Systematic Toxoplasma screening is recommended for all pregnant women, as early in the pregnancy as possible, and is based on serology to assess for the presence of *T. gondii* IgM antibodies. If detected, infection is treated with pyrimethamine and sulfadiazine, which kill the replicating tachyzoite stage of the parasite (239). Since pyrimethamine may be teratogenic in early pregnancy, it is only recommended after 18 weeks of gestation (240). These treatments are highly effective for prevention of congenital *T. gondii* infection, and have shown to reduce incidence of congenital infection up to 60% as compared to historical controls without treatment (240). However, if congenital infection does occur then newborns are also offered these treatments. One of the main drawbacks to the current treatment approach is that *T. gondii* has multiple stages in its lifecycle and the drugs have less efficacy against the slow dividing bradyzoites (239).

### Protective Immunity

Immunological control of *T. gondii* infection requires both innate and adaptive immune responses. A TH1 response through toll-like receptor (TLR) sensing of the parasite in tissues followed by IL-12 and IFNγ production are critical for control of cyst formation (241). Also, dendritic cells and neutrophils are important to parasite control as depletion of these immune cells results in an increased burden of cysts and downstream health complications (242, 243). CD4+ and CD8+ T cell responses and a robust antibody response including IgM, IgA, and IgE antibodies develop by 2 weeks post infection, suggesting an essential role for T cells, mucosal antibodies, and/or extracellular antibody effector functions (234). The importance of T cell responses is demonstrated in HIV patients, where there is a lack of ability to control the infection in patients with reduced CD4+ T cells and also in mouse models lacking CD4+ T cells (244, 245). Although reinfection can occur, a prior exposure to this pathogen and immune response limits spread of the parasite throughout the body. Given that natural immunity does not prevent reinfection, a vaccine would have to provide more robust immunity than natural infection to be effective.

### Vaccine Design Considerations

The multiple life stages of parasites are particularly complex for vaccine design, as these pathogens change substantially over the course of infection. Thus far, there are no licensed vaccines to prevent parasitic diseases, and treatments have been largely effective given their low rates of mutation as compared to viral and bacterial pathogens (246). A vaccine may require targeting of important immune responses required for prevention of infection such as MHC class I restricted CD8+ T cells and IFNγ (247).

In pregnant women prior immunity to *T. gondii* is not sufficient to prevent transmission to the fetus although transmission and severity of injury to the fetus is reduced (248). Vaccine development has been ongoing for the last 30 years, with a focus on prevention of tissue cyst development and vertical transmission in livestock and felines, which are the primary sources of human exposure to the pathogen (249). Blocking the pathogen prior to cyst formation is critical as cysts shield parasites from immune detection and responses, and thereby enable infection and transmission. Thus, parasite control mediated by vaccine-elicited immunity is more feasible before to cyst formation. An example of this approach is the licensed Toxovax® vaccine for sheep. Toxovax® is a live attenuated vaccine consisting of the S48 strain of tachyzoites that reduces abortion rates in sheep and tissue cyst formation (250). However, the disadvantage of Toxovax® is its short shelf life, and incomplete protection. Due to its limited application in breeding ewes, the primary focus of Toxovax® is to improve livestock yields and not for prevention of congenital toxoplasmosis in people.

### How Close Are We

The strategy to vaccinate livestock and household pets is a novel approach to preventing congenital infections, and depends upon high levels of implementation, as pockets of unvaccinated pets and livestock can endanger pregnant women and newborn health. Importantly, this strategy may not be effective in low-income countries where disease burden is greatest due to economic constraints of purchasing vaccines for livestock with limited household income (251). Given the low rates of congenital transmission and options for screening and treatment, development a human vaccine for *T. gondii* continues to be low priority for research. In place of a vaccine, increasing access to screening and drugs may be a more urgent and effective alternative.

## Syphilis

Syphilis, a sexually transmitted infection caused by the spirochete bacterium *Treponema pallidum*, infects 36 million people globally and annually impacts 988,000 pregnancies that result in 661,000 cases of congenital syphilis (CS) per year worldwide (252). Mother to child transmission is the second leading cause of still birth and miscarriage worldwide. Of pregnant women with untreated syphilis globally, 53% have an adverse birth outcome, with 16% of infants with clinical disease (2). Transmission can occur any time during pregnancy. While penicillin can treat this disease, there is rise in newborn infections, mostly due to low coverage of antenatal screening and rising numbers of maternal infections in the general U.S. population (1, 2).

Despite growing recognition of the need to bolster public health interventions and develop a complementary vaccine strategy, there is a lot that remains unknown about the biology of *T. pallidum* and immunity required to protect against it. An important reason to complement these implementation challenges with more urgent vaccine development efforts is due to the rise of antibiotic resistant syphilis strains, suggesting that alternative approaches to penicillin will soon be required (253). In the absence of longstanding syphilis vaccine research, maternal immunity may provide clues as to the immunity required for protection. It is known that mothers who are later in their infection, are substantially less likely (10%) to transmit syphilis to their fetus as compared to those in early infection (40–70%) (254). This indicates that development of adaptive immunity over the course of infection offers substantial protection from congenital disease, and a vaccine could elicit similar types of immunity. Intensive research on the genetic variability across syphilis strains and protective immunity must take place before developing a vaccine to prevent CS.

### Current Guidance

Congenital Syphilis is highly treatable using antibiotics, benzathine penicillin G (255). The CDC mandates syphilis screening at the first prenatal visit, and recommends follow-up screening in early third trimester and delivery in higher risk populations. Syphilis is diagnosed by treponemal or non-treponemal serologic tests which detect syphilis antibodies against these different life stages for the bacterium. Since these tests may have lower sensitivity in early infection, the diagnosis also consists of evaluation of sexual history and exclusion of other diseases. Once diagnosed in pregnancy, 1–3 doses of penicillin (2.4 million units total) are administered intramuscularly to treat the disease. While this is considered curative for the fetus as well, upon birth, the infant also undergoes syphilis serology testing and may receive the treatment.

Despite treatment availability, the majority of congenital infections in high resource settings like the US occur because the mother is unaware of her infection status, due to lack of testing and difficulty in diagnosing low grade early infections (256, 257). Lesions from primary infections can be painless and difficult to detect, whereas secondary and tertiary stages of syphilis can persist without symptoms or be misdiagnosed due to the non-descript nature of symptoms. This leads to missed opportunities for detection and treatment. Moreover, policies for implementation of screening is a critical issue. For example, a 2016 study identified that 6 US states did not require prenatal screening for syphilis at the first visit (257).

Meanwhile, in low- and middle-income countries syphilis remains at endemic levels due to lack of access to antenatal care visits, missed screening at these visits, and increase in prevalence among key populations such as men who have sex with men and female sex workers (1, 258, 259). Additionally, there are global drug shortages of benzathine benzylpenicillin, the treatment for syphilis which has been placed on the essential medicines list by the WHO. Projections of unmet drug need for 30 high burden countries indicate a doubling of penicillin required for mothers and infants if screening guidelines were met for 95% of the population (260). Fulfilling this need would prevent 95,938 adverse birth outcomes and 37,822 stillbirths (260). Therefore, despite effective diagnostic and treatment options, CS has not been controlled in low or high resource settings due to persistent structural and implementation barriers.

### Protective Immunity

*T. pallidum* infection occurs in the mucosa or skin following sexual contact. Spirochetes directly attach and replicate below the skin, and subsequently spread through the blood and lymphatics (261). Cellular immune infiltrates are seen at the sites of replication (262), after sensitization T cells respond to outbreak infections with a delayed type hypersensitivity response, which is associated with clearance of the bacterium (263). Antibodies are also thought to control infection. Following primary infection, high levels of IgG is detected against *T. pallidum* proteins, which are important for opsonization and phagocytosis of the bacterium by macrophages (264). Until 2018, studies of *T. pallidum* have been hindered by an inability to culture this organism long-term *in vitro*; this technology now enables improved evaluation of immunity to this pathogen (265).

Proof of concept for vaccination was shown in 1973 where rabbits were vaccinated with irradiated bacteria 60 times in 37 days and were fully protected from challenge for at least a year from the homologous bacterium strain (266). This work showed that antibodies directed toward native epitopes on the outer membrane of virion surface would be sufficient for protection. However, IgG elicited from infection with one strain is less protective against heterologous strains, suggesting that vaccine design must account for diversity across strains.

Currently, the primary focus for vaccine development is identifying appropriate immunogens. This research has led to characterization of outer membrane (OM) proteins of the bacterium. Identification of OM structures is complicated in *T. pallidum* due to few proteins in the OM and fragility of the bacterium, which makes isolation of OM proteins difficult (261). Advances in bioinformatics have increased the identification of the outer membrane proteins, such as TrpK and reinvigorated funding for identifying a vaccine for Syphilis (267).

### Vaccine Design Consideration

A vaccine to prevent CS should go hand in hand with key public health interventions to improve screening and treatment in pregnancy. Given the population level morbidity, implementation challenges, and high sexual transmission rates, the target vaccine to prevent CS would not only elicit protective immunity prior to pregnancy but also minimize transmission. Therefore, the target population would be men and women prior to sexual debut. Since primary infection in pregnancy leads to higher rates of congenital transmission than later in the course of infection, the target syphilis vaccine will need to protect seronegative populations.

Given the challenges with culturing *T. pallidum* and making large quantities for inactivation or attenuation, a strategy of using bacterial antigens as vaccines is particularly promising. Similar to the pneumococcus, meningitis, and *Haemophilus influenzae* type B vaccines, these bacterical antigens can be presented as a conjugate vaccine with optimized carrier and adjuvants. Additionally, conjugate vaccines have a strong safety profile for administration to pregnant women at all stages of pregnancy (255).

### How Close Are We

Priority for vaccine development and syphilis research has been low due to the availability of penicillin as a treatment for syphilis. However, implementation challenges associated with timely screening and treatment have led to a failure to eliminate CS thus far. Importantly, resistant strains threaten the effectiveness of this approach in the future. Development of a vaccine would be extremely beneficial for prevention of syphilis related still births and congenital infection. A syphilis vaccine may also contribute to control of HIV infection as syphilis infection is associated with enhanced HIV transmission (237).

## Zika Virus (ZIKV)

Zika virus (ZIKV) is the most recently discovered congenital pathogen and transmits from mother to child during pregnancy in ~1 out of 10 ZIKV-infected pregnancies (268–270). The congenital route of transmission for ZIKV was first identified during the 2015–2016 outbreak in the Americas (271, 272). However, ZIKV may spread sexually, and primarily via the ubiquitous *Aedes* genus mosquito vector (273). In healthy individuals, ZIKV infection may be asymptomatic or cause a mild short-lived febrile disease with rashes, conjunctivitis, and arthralgia (274). Whereas, Congenital Zika Syndrome (CZS) is much more severe outcome and characterized by an array of conditions including neurodevelopmental disabilities, microcephaly, visual impairments, motor delays, and reduced mobility due to muscle contractures (268, 275, 276). These conditions cause lifelong disability. In one cohort, one third of infants born to mothers with ZIKV-infection in pregnancy presented with below average scores for neurodevelopment, and abnormal vision, hearing, and language function by the age of two (276). There have also been reports of cardiac defects, and development of microcephaly and autism in early life (276). It is estimated that a single CZS case would result in $100 million in healthcare costs in the United States (277). The burden of Zika disease on congenitally infected newborns has motivated intensive research and unprecedented collaborations to publishing findings rapidly for vaccine development.

Though the ZIKV outbreak of 2015–16 has subsided, the virus may re-emerge after years as the population grows susceptible to sustain transmission, like other flaviviruses. The virus is capable of infecting urban primate populations in South America that could serve as a reservoir for future outbreaks, or alternatively buffer outbreaks through immunity (278–280). The previous ZIKV outbreak led to microcephaly in 11,000 newborns in Brazil and decline in fertility rate on a national level (281, 282). If the virus were to return after years, these devastating consequences for people and countries must be prevented by a vaccine or intervention that is effective in pregnancy.

### Current Guidance

Presently, there are no licensed vaccines or antivirals for the prevention or treatment of Zika virus in pregnancy, the time period of greatest risk to the fetus. Given the lack of therapeutic options and the lifelong burden of disability, the primary guidance is to prevent ZIKV-infection in pregnancy. Pregnant women are advised to avoid travel to ZIKV-endemic areas, minimize mosquito exposures, and protect against sexual transmission during pregnancy (283). Condom use or abstinence from sexual contact after travel to endemic regions is recommended for up to 3 months with a male partner, due to prolonged persistence of ZIKV RNA in semen (283). Moreover, community mosquito control for arbovirus outbreaks in densely populated areas may help to reduce transmission but does not offer complete protection, as mosquito populations may be missed and rebound (284).

Pregnant women living in outbreak areas with symptoms or travel exposure history may be tested via a PCR test for the presence of viral RNA or presence of ZIKV-specific IgM for up to 12 weeks from exposure. While the PCR diagnostic is most effective in acute infection, the CDC supports longer testing in pregnancy as ZIKV may persist up to 3-times longer in pregnant as compared to non-pregnant women (285, 286). After birth, infants suspected of *in utero* ZIKV exposure may be tested for ZIKV RNA or IgM, even though the molecular test is most effective within 10–14 days of acute infection and there is no reliable infant diagnostic (287). While ZIKV RNA and infectious virus has been reported in breast milk, postnatal transmission via this route has not been reported and thus breast feeding of newborns by ZIKV-exposed mothers continues to be recommended by the WHO (288).

### Protective Immunity

Knowledge on immunity required to protect against ZIKV-infection comes primarily from interferon knockout mouse models, non-human primate models, and observational cohorts of mothers and infants sampled during the recent outbreak (289–291). In the aftermath of 2015–16 outbreak, several vaccine candidates were developed and tested in mice and NHPs, including an envelope (E) and pre-membrane (prM) viral protein encoded as a DNA vaccine and mRNA vaccine, purified inactivated virus, live attenuated vaccine, and adenovirus vectored E and prM. All candidate vaccines protected non-pregnant NHP and interferon knockout mice from challenge (292–296). Moreover, neutralizing mAbs and purified immunoglobulin from immunized monkeys protected non-pregnant NHPs from ZIKV challenge, suggesting that high titers of neutralizing antibodies alone may serve as a correlate of protection (292, 295). Based on these promising findings, the DNA vaccine encoding E and prM viral proteins was tested in Phase I clinical trials and shown to be safe and immunogenic in humans (297). Yet, in pregnant NHPs, the leading DNA vaccine candidate and neutralizing monoclonal IgG therapeutics have failed to provide sterilizing maternal and fetal immunity with cases of fetal viremia and brain pathology despite vaccination (298, 299).

As an alternative to the vaccine candidates based on structural viral proteins, such as E, vaccine designs with non-structural protein 1 (NS1) have also been tested in the mouse model. The NS1 based candidates demonstrate reduced viral load and improved survival in mice but cannot offer sterilizing immunity as NS1 is not externally displayed on the virion for neutralization (300, 301). In improving vaccine design to protect in pregnancy, an E and NS1 combined vaccine design may be considered in the future.

Studies of human responses after ZIKV infection indicate rapid elicitation of plasmablasts and establishment of a ZIKV-specific memory B cell population, supporting the protective role for B cell immunity and ZIKV-specific antibodies (302, 303). Furthermore, analyses of human T cell peptide epitopes shows that ZIKV specific CD8+ T cells react more to structural proteins whereas CD4+ T cells have a greater response to non-structural viral proteins (304–306). Additionally, genetic variability across ZIKV strains is not likely to be critical to vaccine design as ZIKV is one serotype, and neutralizing antibodies against one strain protects against the other (307, 308).

Innate immune host variability may also modulate disease severity in pregnancy and capacity to induce protective maternal immunity with vaccines. Through the mouse models and *in vitro* studies of placental explants and primary human trophoblasts it has been found that interferon responses mediate pathogenesis at the maternal-fetal interface. While type I IFNα/β inflammatory responses mediate an antiviral response they also cause placental damage in the setting of ZIKV infection, whereas type III IFNγ1 restricts ZIKV (309, 310). In addition, complement may inhibit ZIKV replication in an antibody-dependent and independent manner, which may also be altered during pregnancy (311–313). Progress thus far indicates a need to evaluate active and passive vaccination strategies against Zika in a pregnancy model of infection to design optimal candidates.

### Vaccine Design Considerations

All vaccine strategies have an emphasis on prevention of congenital infection, since this defines the primary disease burden. The target population for a vaccine is both male and female populations prior to reproductive age, with a primary goal of a vaccine should be to minimize risk of infection in pregnancy by eliciting protective immunity that is effective in pregnancy. Moreover, vaccinating both men and women will disrupt transmission across populations and reduce likelihood of viral exposure in pregnancy, either through mosquitoes or sexual contact.

A key concern about ZIKV vaccine development is cross-reactivity of antibody responses with co-endemic Dengue (DENV) viruses, which are antigenically similar (314). Cross-reactive antibodies that target conserved epitopes across these viruses have the potential to mediate antibody-dependent enhancement of subsequent viral infection (315–317). Thus far, cohort studies suggest that prior DENV leads to reduced risk of ZIKV infection (318, 319). Moreover, in NHP's prior ZIKV did not adversely impact DENV pathogenesis, though further epidemiologic data is needed (307, 320, 321). It is known that vaccine-elicited IgG and flavivirus specific IgG are efficiently transferred across the placenta, therefore the impact of these antibodies on fetal and neonatal health must be considered in vaccine development (322).

### How Close Are We

Currently, vaccines candidates cannot be assessed for efficacy without on-going transmission. Therefore, the emphasis for vaccine development efforts is on research using animal models and observational cohorts. When Phase II and III efficacy testing is feasible, a successful candidate will have to demonstrate vaccine efficacy in a combination of pregnant and non-pregnant populations. Moreover, as with Ebola vaccine trials, ethical and innovative vaccine trial designs will be necessary in order to assess a vaccine candidate in the context of an outbreak (323, 324). Due to the requirement to assess Zika vaccine candidates within outbreak settings, it is possible that evidence for licensure may be derived not only from vaccine trials but also optimal animal models.

## Conclusion

Evaluating progress in vaccine development for congenital and perinatal pathogens reveals a varied landscape of priorities based on global disease burden and availability for alternative prophylactic or treatment options. Intensive research investments and progress is characterized by an understanding of the features of protective immunity within animal models and with precedence of clinical vaccine trials that inform on-going research gaps. For Zika, CMV, HSV, and syphilis, the optimal active vaccination would be delivered prior to pregnancy like the rubella and varicella vaccine due to risk of transmission across every stage of pregnancy. These vaccines will need to elicit long lasting sterilizing immunity to prevent infections during pregnancy. Whereas, immunologic approaches for elimination of HIV mother to child transmission have revealed a series of complexities with viral diversity and escape from immunity, leading to increasingly complex passive and active vaccine components that remain to be tested. It is possible that passive maternal immunization could curb vertical transmission rates for ZIKV and HIV, as with Hepatitis B, yet evidence suggests that this will not likely be an effective strategy for CMV and HSV vaccines which may require protective cellular responses in addition to antibody responses. In contrast to these strategies that will be optimal before pregnancy, the GBS vaccine candidates are specifically designed for administration in pregnancy to promote transplacental transfer of antibodies and prevent early and late onset of neonatal disease.

Moreover, vaccine testing approaches also vary. Syphilis and toxoplasma present a unique situation for vaccine development since effective treatment options are available but are rendered ineffective due to implementation challenges. These barriers emphasize the role of public health interventions in promoting pediatric health. Indeed, the availability of a treatment supports vaccine development and testing in seronegative populations as there are well-established standards of care for even the placebo group upon detection of disease. In comparison, a maternal HIV vaccine would only be administered to actively infected individuals and therefore a novel vaccine candidate must demonstrate efficacy in the presence of the ART as standard of care. Whereas for CMV vaccines, these can be tested in seronegative or seropositive populations. Finally, ZIKV vaccines cannot be tested without transmission in the population, which suggests roles for optimal animal models to guide vaccination strategies.

To make further progress on these vaccines for congenital and perinatal infections and protect newborns, it is important to evaluate the vaccine candidate in the relevant setting of mother to child transmission, taking into account the role of pregnancy on immunity and the timing of screening and disease detection. This will allow for more effective translation of vaccine strategies for maternal and newborn health.

## Author Contributions

TS and SP substantially contributed to the conception and design of this work and iteratively reviewed sections. TS led the writing process. TS, CO, KL, SV, AN, and SP provided intellectual contributions, offered interpretations and synthesis of the literature, and supported in drafting and revising this work. SP provided approval for publication of this content. All authors contributed to the article and approved the submitted version.

## Conflict of Interest

SP is serving as a consultant for vaccine programs at Merck, Pfizer, and Moderna. The remaining authors declare that the research was conducted in the absence of any commercial or financial relationships that could be construed as a potential conflict of interest.
